# Reflection on the RANZCP position on the adverse effects of psychotherapy

**DOI:** 10.1177/10398562241280362

**Published:** 2024-09-04

**Authors:** Stephen Allison, Jeffrey CL Looi, Steve Kisely, Tarun Bastiampillai

**Affiliations:** College of Medicine and Public Health, 1065 Flinders University, Adelaide, SA, Australia; and; Consortium of Australian-Academic Psychiatrists for Independent Policy and Research Analysis (CAPIPRA), Canberra, ACT, Australia; Academic Unit of Psychiatry and Addiction Medicine, 2219 The Australian National University School of Medicine and Psychology, Canberra Hospital, Canberra, ACT, Australia; and; Consortium of Australian-Academic Psychiatrists for Independent Policy and Research Analysis (CAPIPRA), Canberra, ACT, Australia; School of Medicine, 1974 The University of Queensland, Princess Alexandra Hospital, Ipswich Road, Woolloongabba, Brisbane, QLD, Australia; and; Consortium of Australian-Academic Psychiatrists for Independent Policy and Research Analysis (CAPIPRA), Canberra, ACT, Australia; Department of Psychiatry, Monash University, Clayton, VIC, Australia;; College of Medicine and Public Health, 1065 Flinders University, Adelaide, SA, Australia; and; Consortium of Australian-Academic Psychiatrists for Independent Policy and Research Analysis (CAPIPRA), Canberra, ACT, Australia

**Keywords:** psychotherapy, adverse effects, monitoring and evaluation of therapy

## Abstract

**Objective:**

In this perspective, we investigate how the Royal Australian and New Zealand College of Psychiatrists’ (RANZCP) position statement on psychotherapy takes the potential for adverse effects into account.

**Conclusions:**

Psychotherapy has two critical outcomes – efficacy and adverse effects. Evidence-based psychotherapy is significantly more effective than care-as-usual for about one in 10 psychotherapy patients. However, a similar proportion also reports adverse effects. Despite this, the RANZCP position statement on psychotherapy focuses on efficacy with minimal discussion of the adverse effects. This is an oversight because psychiatrists have legal and ethical obligations to consider the adverse effects as well as the benefits of any treatment. We therefore reflect on the RANZCP’s six recommendations in light of the adverse effects of psychotherapy.

*‘Facing it, always facing it, that’s the way to get through. Face it.’ - Captain McWhirr, Typhoon, by Joseph Conrad*

Psychotherapy is central to psychiatry. The most recent position statement on psychotherapy was released by the Royal Australian and New Zealand College of Psychiatrists (RANZCP) in 2021.^1^ The question that led to this perspective was ‘How does this statement take into account the adverse effects of psychotherapy (hereinafter PAEs) when making recommendations on psychotherapy?’

We found that the RANZCP position statement focuses on the well-established benefits of psychotherapy with minimal comment on the potential adverse effects. However, ‘non nocere’ (do no harm) is one of the most important ethical rules in psychiatry. Psychotherapeutic conversations can and do have adverse effects. ‘*For most patients, it can be burdensome to speak about insufficiencies of oneself, to be confronted with painful memories, to be exposed to threatening situations, or to be subject to treatment demands’.*^2^ Psychiatrists are obliged to inform patients about these potential harms when gaining consent for therapy.^2^

We therefore provide a perspective that aims to advance the recognition of PAEs and their implications for RANZCP psychotherapy training, clinical practice, and research. Our perspective is structured to: (1) define PAEs, (2) summarise the qualitative and quantitative studies of PAEs, and (3) provide evidence-based reflections on the six RANZCP recommendations on psychotherapy in light of recent reviews of PAEs (Box 1).^3–5^

## Defining the adverse effects of psychotherapy

The World Health Organization (WHO) defines the term ‘adverse effects’ as the subjectively unpleasant consequences of any treatment that were not the intended goal.^6,7^ Adverse effects vary in the degree of physical, psychological or social suffering, and associated impairment in functioning, and are measured on a continuum.^6^ Harm is determined by the severity and duration of adverse effects and any resulting treatment implications.^6^

We therefore used these concepts as reported by the patient or observed by close family members and/or the treating therapist to correctly applied evidence-based psychotherapy such as psychodynamic therapy or cognitive behavioural therapy (CBT). Since the outcomes of psychotherapy are inherently subjective (e.g. improved or worsened mood), patient reports of PAEs are the most important measure although close family members, friends, colleagues, and the therapist may be affected by changes in the patient. Other than the particular type of psychotherapy offered, PAEs are related to the therapeutic skills of the therapist, the quality of the two-person therapeutic relationship, and the patient’s presentation or personality.^8^ PAE rates are probably higher in the real world than in clinical trials with manualised treatments and close supervision because of varying levels of therapist skills, training, and adherence to therapy principles.

## Qualitative and quantitative studies of adverse effects

Patient lived experience informs descriptions of PAEs, given they have unique knowledge about their own life journey and what constitutes recovery.^9^ Psychotherapy requires epistemic trust that the therapist’s knowledge is credible and applicable to the patient’s life. Consequently, negative experiences are associated with epistemic vigilance or mistrust of the therapist’s competence or benevolence.^3,9^ Qualitative studies indicate that patients often attribute negative experiences to therapist misbehaviour or poor treatment fit.^3^ These, in turn, are associated with the following:

• Increased problems after therapy
• Fear of the therapy process
• Loss of motivation or hope
• Unpleasant feelings during therapy
• Negative cognitions aroused in therapy.^3^

Dynamic formulations of PAEs have a similar focus on the therapeutic relationship where a patient forms a dependent transference relationship with their therapist, thereby incurring a loss of agency and capacity to independently problem-solve and regulate their emotions, leading to worsening symptoms.^10^ In some longer-term therapies, there is the potential for regression, greater self-absorption, and adoption of the sick role with the risk of further PAEs during the termination of treatment.^10^ PAEs are considerably less likely when therapy is viewed as a shared endeavour with patients and therapists undertaking a collaborative journey that results in enhanced patient self-agency.^9^

PAEs are one of the two critical outcomes in randomised controlled trials (RCTs) of psychotherapy.^3,4^ The first critical outcome is measuring whether therapy works (efficacy or effectiveness), and the second critical outcome is tolerability, that is, whether a patient can withstand any PAEs so therapy has a meaningful impact.^11^ As with any psychiatric treatment, the benefits of psychotherapy should outweigh the adverse effects. Current RCT-level evidence demonstrates that evidence-based psychotherapy is effective for about one in 10 psychotherapy patients compared to care-as-usual controls, while RCT reports also indicate that a similar proportion experience PAEs in making these significant gains.^4,12^

All RCTs of psychotherapy should therefore measure clinically significant improvement and PAEs in the same detail in both intervention and control groups.^4,5^ In particular, comprehensive patient interviews with independent assessors provide more in-depth descriptions of PAEs.^7^ To meet the independence criterion, PAE assessors should be unaffiliated with the clinical trial and not biased for or against the particular type of psychotherapy.^7^

As with the adverse effects of medication, patients may experience unpleasant consequences of psychotherapy even if their overall clinical state improves.^2^ PAE rating scales measure patient and observer reported harm.^13^ Over 1 in 10 patients or therapists have reported PAEs in the following domains:

• Increased problem complexity
• Therapy dependency
• Therapy burdensome
• Symptom deterioration
• New symptoms
• Stigmatisation.^14^

## Evidence-based reflections on the six RANZCP recommendations

### Recommendation 1. Psychotherapy must be embedded in training for all psychiatrists

We reflect on the RANZCP’s recommendations on psychotherapy, in light of recent reviews of qualitative and quantitative evidence on PAEs.^3–5^ The first recommendation is that ‘*Psychotherapy, as a key component of every psychiatric treatment, must be embedded in training for all psychiatrists’* (Box 1).^1^

This recommendation is based on the efficacy of psychotherapy. However, therapists have long recognised that psychotherapy also holds dangers for some patients, depending on complex interactions between the patient, their therapist and the psychotherapeutic approach.^8,10^ Because psychotherapy is a keystone of every psychiatric treatment, *all* patients need information on both the effectiveness of psychotherapy and the PAEs.^2^ This forms the basis for informed consent and shared decision-making. If the possibility of PAEs emerging during treatment is not specifically addressed, patients and therapists tend to misattribute PAEs to life circumstances or the underlying psychiatric condition.^2^ It requires therapeutic skill to inform patients about the realistic possibility of PAEs, while avoiding nocebo effects due to excessively negative expectations of the treatment. However, routine outcome measurement of effectiveness and PAEs can enhance the therapy process.^14^

**Box 1.** RANZCP recommendations on psychotherapy

The RANZCP recognises the importance of psychotherapy in the provision of mental health care and is committed to training psychiatrists in psychotherapy and supporting psychiatrists to use psychotherapeutic treatment modalities in their practice. Psychiatrists value the lived experience of people with mental disorder and the healing potential in the relationship between those being treated and the psychiatrist. In acknowledgement of these facts, the RANZCP takes the following position:
(1)Psychotherapy, as a key component of every psychiatric treatment, must be embedded in training for all psychiatrists
(2)Psychiatrists and psychiatry trainees interested in further study of psychotherapy should be supported to do so through advanced training. In addition, further study and training should be available at all levels in psychotherapy
(3)Psychotherapy conducted by psychiatrists should be valued as a core treatment modality in public and private practice. It should be funded and widely available to the population with conditions that would benefit from this treatment across all service settings
(4)Psychiatrists have medical training which, when complemented by the biopsychosocial perspective on mental health, facilitates a holistic approach to mental health care. Psychiatrists are therefore ideally placed to ensure that people with mental disorder are provided with a balance of psychotherapy and medication treatment modalities appropriate to their needs
(5)Psychotherapy can provide benefits which other treatment modalities cannot and therefore psychiatrists should always consider the value of psychotherapy when providing treatment
(6)There is a need for continuing research into the efficacy and potential side effects of psychotherapy including on the specific types and elements of psychotherapy in relation to benefits in specific conditions, as well as the cost-effectiveness of different types of psychotherapy for different conditions

RANZCP. Psychotherapy conducted by psychiatrists. Position statement #54. Melbourne, Australia: Royal Australian and New Zealand College of Psychiatrists, 2021.

### Recommendation 2. Further study in psychotherapy should be supported

Given that the RANZCP recommends advanced training in psychotherapy, this should be based on evidence-based principles. As a result, training should also include the recognition, evaluation, documentation, and management of PAEs.^2^ To facilitate this process, the brief UE-PT (unwanted events in the view of patient and therapists) scale has been developed for completion at regular intervals during the course of psychotherapy.^13^ Interestingly, patients tend to react positively to therapists suggesting the use of the scale during therapy.^14^ This is because discussion of potential PAEs can strengthen rather than endanger the therapeutic alliance as the patient and therapist are engaged in a cooperative venture that builds interpersonal trust.^9^ Box 2 provides the series of areas that patients and therapists rate for severity and discuss whether or not any unwanted events are caused by therapy and can be regarded as PAEs.

**Box 2.** Indicators of possible psychotherapy adverse effects

(1)Worsening of presenting symptoms
(2)New symptoms appearing
(3)Increased complexity of presenting problems
(4)Therapy sessions are very stressful
(5)Problems with the practical demands of therapy
(6)Difficult relationship between therapist and patient
(7)Dependence on therapy
(8)Deterioration in close family (partner and children) relationships
(9)Relations with the wider family (parents and other relatives) deteriorate
(10)New problems with friends and neighbours
(11)Problems emerging at work (with superiors, colleagues, and work performance)
(12)Unsatisfactory progress of therapy
(13)Therapy sessions becoming more frequent
(14)Negative developments in life
(15)Therapy regarded negatively by others

Adapted from: Linden M. Parallel recording of psychotherapy side effects by patient and therapist in routine as well as training therapies by means of the ‘UE-PT scale’. *Psychosoz Med. Rehabil* 2021; 116: 63–70.

### Recommendation 3. Psychotherapy should be widely available to the population

Although the RANZCP recommends that psychotherapy should be widely available in public and private practice for patients with a variety of mental conditions, this may have negative as well as positive effects. For instance, a large survey of psychotherapy patients found one in 20 reported persistent PAEs, while another reported that 14% of patients experienced persistent PAEs after psychotherapy for depression or anxiety.^15,16^

Programmes to expand access to psychotherapy should therefore take these risks into account. This might include measuring and publishing patient and therapist ratings of PAEs and whether clinical deterioration is attributed to psychotherapy or other factors. Patients need information about their chances of improving or deteriorating after psychotherapy, based on these data.

### Recommendation 4. Psychiatrists providing a balance of psychotherapy and medication

The RANZCP highlights that psychiatrists are trained in the use of psychotherapy and pharmacotherapy, which ensures that their patients are provided with the right balance of treatment. Adding psychotherapy to medication may improve treatment outcomes for patients with severe psychiatric conditions.^1,12^ For example, combined psychotherapy and antidepressants are recommended as the treatment for severe depression, based on clinical and cost-effectiveness.^17^

In addition to maximising the benefits of combined treatment for severe depression, psychiatrists are also able to minimise the combined adverse effects of psychotherapy and antidepressants. The biological adverse effects of antidepressants include weight gain, sedation, and effects on sexual function, while PAEs include increased problem complexity and symptom aggravation due to psychotherapy.^3,4,17^ Patients receiving combination treatment for severe depression can experience various interactive combinations of these adverse effects, for example, increased problem complexity and sexual dysfunction. Psychiatrists should detect these psychotherapy and pharmacotherapy adverse effects and adjust treatment to alleviate them.

### Recommendation 5. Psychotherapy providing unique benefits

The RANZCP notes that psychotherapy provides benefits that other treatment modalities cannot. For example, the treatment of choice for severe personality disorder is psychotherapy, because pharmacotherapy is not effective for the core features.^18^ There is a signal that psychotherapy reduces the risks of self-harm and suicide-related behaviour associated with severe personality disorder.^18^ However, research into PAEs has been neglected in this population, and future trials should therefore compare intervention groups with care-as-usual controls on relevant measures such as difficulties in the therapeutic relationship and reawakened traumatic memories. The expectation is that psychotherapy would be superior to care-as-usual on PAEs, but this benefit needs to be quantified.

### Recommendation 6. The need for continuing research into psychotherapy

Finally, the RANZCP recommends further empirical research into the efficacy, cost-effectiveness, and PAEs of specific types of psychotherapy for different conditions. Given the gaps in the evidence that we have identified on the PAEs associated with popular psychotherapies such as psychodynamic therapy and CBT, clinical trials of these psychotherapies should systematically measure PAEs as well as efficacy and cost-effectiveness.^5^ Further empirical research is also needed on optimising the process of informed consent and shared decision-making by discussing PAEs.^19^

## Strengths and limitations

The major weakness of this Perspective is the limited available information on PAEs on which to base our conclusions. However, a strength is that it also provides the first preliminary discussion of the RANZCP recommendations on psychotherapy in light of the available qualitative and quantitative evidence on PAEs.^3–5^ Psychiatrists have legal and ethical obligations to consider the adverse effects of any treatment and inform their patients accordingly.^2^ Greater recognition of the potential for PAEs could therefore contribute to RANZCP psychotherapy training, clinical practice and research. Minimising PAEs though early recognition is a possible means of improving patient outcomes from psychotherapy above the current ceiling for treatment efficacy.^12^ It is also foundational to the ethical practice of psychotherapy.^2^
